# Engineered Extracellular Vesicles in Treatment of Type 1 Diabetes Mellitus: A Prospective Review

**DOI:** 10.3390/biomedicines10123042

**Published:** 2022-11-25

**Authors:** Alok Raghav, Hamid Ashraf, Goo-Bo Jeong

**Affiliations:** 1 Multidisciplinary Research Unit, Sponsored by Department of Health Research, Ministry of Health and Family Welfare, GSVM Medical College, Kanpur 208002, India; 2 Rajiv Gandhi Centre for Diabetes and Endocrinology, J.N. Medical College, Aligarh Muslim University, Aligarh 202002, India; 3 Department of Anatomy and Cell Biology, College of Medicine, Gachon University, 155 Getbeol-ro Yeonsu-gu, Incheon 21999, Republic of Korea

**Keywords:** type 1 diabetes, insulin, immunosuppression, extracellular vesicles, tolerogenic nanoparticles

## Abstract

Insulin replacement is an available treatment for autoimmune type 1 diabetes mellitus (T1DM). There are multiple limitations in the treatment of autoimmune diseases such as T1DM by immunosuppression using drugs and chemicals. The advent of extracellular vesicle (EV)-based therapies for the treatment of various diseases has attracted much attention to the field of bio-nanomedicine. Tolerogenic nanoparticles can induce immune tolerance, especially in autoimmune diseases. EVs can deliver cargo to specific cells without restrictions. Accordingly, EVs can be used to deliver tolerogenic nanoparticles, including iron oxide-peptide-major histocompatibility complex, polyethylene glycol-silver-2-(1′H-indole-3′-carbonyl)-thiazole-4-carboxylic acid methyl ester, and carboxylated poly (lactic-co-glycolic acid) nanoparticles coupled with or encapsulating an antigen, to effectively treat autoimmune T1DM. The present work highlights the advances in exosome-based delivery of tolerogenic nanoparticles for the treatment of autoimmune T1DM.

## 1. Introduction

Type 1 diabetes mellitus (TIDM), also known as juvenile diabetes or insulin-dependent diabetes, is an autoimmune disorder driven by T cell-mediated destruction of pancreatic β-cells that is often initiated during childhood [1]. The pathogenesis of T1DM is driven by the autoreactivity of T cells activated by alleles encoding human leukocyte antigens (HLAs) [2]. In a recently published meta-analysis, the incidence of T1DM was 15 per 100,000 people and its global prevalence was 9.5% (95% CI: 0.07 to 0.12) [3]. T1DM affects 3–10% of people with HLA risk alleles, with little environmental contribution [4]. In patients with T1DM, the seroconversion phase is characterized by the formation of autoantibodies against insulin-producing cells [5]. In a previously published study, it was found that some of the HLA class II haplotypes were associated with a higher risk of T1DM [6]. A prior study indicated the contribution of “natural apoptosis” or spontaneous cell death of the β-cell population and islet amyloid polypeptide aggregates and viral infection to β-cell death due to auto-antigen production, dendritic cell (DC) activation, and antigen presentation [7]. The discomfort associated with regular subcutaneous injections, episodes of hypoglycemia, and lack of knowledge on the management of T1DM can sometimes result in severe hypoglycemia that can have fatal consequences. Improved and more efficient therapeutic approaches for the management of T1DM that are free of limitations are required.

Nanoparticles (NPs) and extracellular vesicles (EVs) have evolved as breakthrough therapies for the treatment of various diseases. NPs and EVs provide a cell-free approach to disease amelioration with advantages that include reduced harmful effects, site-specific delivery, and stability. EVs and engineered/tailored nanoparticles derived from gold, carbon, metal oxides, or degradable polymers have been demonstrated to have beneficial effects on target cells. Tailored NP and EVs are being exploited for the immunomodulation and treatment of various immune-related diseases [8]. Tolerogenic NPs (TNps) provide immune tolerance by modulating the normal immune response through three approaches: (i) modulating natural tolerogenic processes, (ii) targeting pro-tolerogenic receptors, and (iii) using pharmacological immunomodulators to inhibit tolerogenic immune responses. EVs play a critical role in the modulation of immune responses mediated by their cargo of cytokines, growth factors, and functional microRNAs (miRNAs) via the paracrine effect. EVs derived from neutrophils and macrophages have immunosuppressive and immunostimulatory activities by spreading alloantigens and modulating antigen presentation to T lymphocytes [9]. 

In the present review, we discuss the therapeutic advances and applications of EVs for the treatment of T1DM and EV-based delivery of TNps for the treatment of autoimmune T1DM. Our aim is to provide current research updates on TNp-based therapies in immunomodulation and to reveal different treatment strategies for T1DM. 

## 2. Islet Autoimmunity

T1DM patients have autoantibodies in their serum. These include insulin autoantibodies (IAA), glutamic acid decarboxylase 65 (GADA), islet antigen-2 antigen (IA-2A), zinc transporter 8 (ZnT8A), Imogen 38, pancreatic duodenal homeobox factor 1 (PDX1), chromogranin A (CHGA), heat shock protein 60 (hsp60), pro-pre-insulin, and islet cell antigen-69 (ICA-69) [10,11]. The presence of one major autoantibody in the serum of T1DM patients since childhood poses a lower risk to the patient than the presence of two autoantibodies. Maternal transmission of islet-specific autoantibodies increases the risk of autoimmune diabetes [12]. However, according to another study, the transfer of islet autoantibodies from the mother to fetus does not contribute to fetal cell damage [13]. Genome-wide association study findings revealed that HLA genes account for 50% of the genetic risk of developing T1DM, demonstrating that specific auto-antigen peptides contribute to the pathogenesis of T1DM [14]. In a meta-analysis, non-HLA factors, including polymorphism within insulin variable number of tandem repeats (INS-VNTR), protein tyrosine phosphatase non-receptor type 22 (PTPN22), cytotoxic T-lymphocyte associated protein 4 (CTLA4), interleukin-2 receptor subunit alpha (IL2RA), and increased T cell activation and proliferation contribute to the pathogenesis of T1DM [15,16,17,18]. Postmortem pancreas investigations of T1DM patients at different time intervals revealed the infiltration of CD8+ and CD4+ T cells, macrophages, and B cells, thereby indicating damage to β-cells [19]. CD4+ cells specific for the C-peptide of the proinsulin precursor of insulin have been detected in the pancreas of T1DM patients. Islet autoantibodies are diagnostic biomarkers that differentiate between T1DM and T2DM, which evolve from autoreactive B cells and CD4+ T cell associations.

T1DM alleles DR4, DQ8, and DQ2 increase the genetic risk of developing T1DM in humans [20]. CD4+ T cells stimulate antibody production mediated by B cells, as well as CD8+ T cell-mediated responses, which stimulate islet-resident macrophages [21,22]. CD8+ T cells play an important role, as revealed by Faridi et al. [23], who focused on the generation of novel targets against CD8+ T cells. In another study, IAA in T1DM patients was predominantly composed of IgG1 (83%), IgG4 (42%), and IgG2 (17%) [24]. The detailed mechanism for evading tolerance by CD4+ and CD8+ T cells, which modulate the immunological response in T1DM, remains unclear. Clarifying the mechanism may enable the discovery of novel auto-antigenic targets. 

The primary factors for autoimmune responses are also unclear. However, specific autoantigens processed by antigen-presenting cells (APCs) may be contributing factors in T1DM. These APCs include DCs, macrophages, and B cells present in pancreatic islets. The presentation of these antigens to naïve T cells mediated by HLA generates autoreactive CD4+ T cells that become active and generate cytokines following activation of β-cell-specific cytotoxic CD8+ T cells. This immunological cascade attracts activated T cells to the pancreatic islets and stimulates macrophages and other T cells, which initiate islet cell destruction [25].

## 3. Treatment of Autoimmune T1DM 

Based on clinical trials, autoimmune T1DM can be managed by supplementation with immunosuppressive drugs, such as cyclosporine, azathioprine, and prednisone, during the initial phase of T1D onset, which helps in better management. However, these immunosuppressive drugs bear several limitations and adverse effects, including an increased risk of infection, malignancies, and other clinical complications [26]. To overcome these limitations, safer treatment approaches that are currently being used include teplizumab, rituximab, abatacept, and otelixizumab monoclonal antibodies [26]. Another approach involves the use of antibiotics, such as vancomycin, for the treatment of T1DM-related autoimmune disorder [27]. Notably, immunosuppressive drugs and antibiotics do not treat the underlying autoimmunity and require a specific antigen-specific approach to tackle autoimmune disorders, such as T1DM.

T1DM can be treated using currently available therapies. A previously published study in this direction described the delivery of soluble antigens with GAD, insulin, and proinsulin using different routes of administration, including intraperitoneal, intravenous (i.v.), intranasal, subcutaneous (s.c.), and oral, for immunotherapy in a T1DM murine model [28]. Another study involving non-obese diabetic (NOD) and transgenic mice revealed that the infusion of BDC2.5 via s.c. and i.v. injection effectively protected against autoimmune T1DM [29]. Previously published studies [30,31] also highlighted cell-based treatment approaches for T1DM. The current paradigm of cell-free therapies is based on paracrine factors, including exosomes derived from different cell lineages. The exact mechanism underlying the modulation of the immune system by these exosomes is still not fully understood. However, these exosomes are hypothesized to regulate the immune function of cells, including macrophages, natural killer (NK) cells, B cells, and T lymphocytes. 

## 4. Biology of EVs 

EVs are membrane-bound nanovesicles of endosomal origin inherited from cargo loading characteristics that are released into the extracellular fluids by cells [32]. According to the Minimal Information for Studies of Extracellular Vesicles 2018 (MISEV 2018) guidelines, EVs can be defined as components of the complete secretome secreted by the cell without any specific distinguishable marker to differentiate EV subtypes and their subcellular origin [33]. According to the MISEV 2018 criteria, two types of EVs are secreted by cells: exosomes and microvesicles. These types are differentiated based on the mode of biogenesis, rather than size (Table 1). EV biogenesis is a housekeeping phenomenon of cells that is evident as the inward invagination of the plasma membrane within the cytosol, forming early and late endosomes (LEs). These LEs fuse to form multivesicular bodies (MVBs) that undergo further invagination to form intraluminal vesicles (ILVs) [34]. These ILVs fuse with the plasma membrane of the cell to release exosomes into the extracellular space via exocytosis [28].

Sorting of EVs (exosomes) follows one of two pathways. The first pathway is ESCRT-I (endosomal sorting complex required for transport I)-dependent cargo sorting. This pathway includes the identification and sequestration of ubiquitinated proteins to specific sites of the endosomal membranes. This enables an association between ESCRT subunits I, II, and III, which further initiate the budding process. Budding is terminated by the Vps-4 protein factor, which is involved in the detachment of the ESCRT-III complex from the MVB membrane [34]. The ESCRT-independent mechanism of exosome sorting involves proteins and lipids, such as tetraspanins (CD81) and ceramides [34].

EVs are enveloped in a lipid bilayer anchored with functional proteins on their surface. The proteins include surface proteins, such as cluster of differentiation (CD) and major histocompatibility complex (MHC). The protein content within exosomes depends on the source cells of EVs and the cell stimulus (i.e., the microenvironment) [35]. These cell-derived EVs are a rich source of proteins, including heat shock proteins, cell adhesion proteins, trafficking membrane fusion proteins, tetraspanin membrane proteins, cell signaling proteins, and transcription proteins [34]. Moreover, several lipids and RNAs bearing therapeutic and diagnostic value are also present in EVs [34].

## 5. Tolerogenic Role of EVs

**Figure 1:**
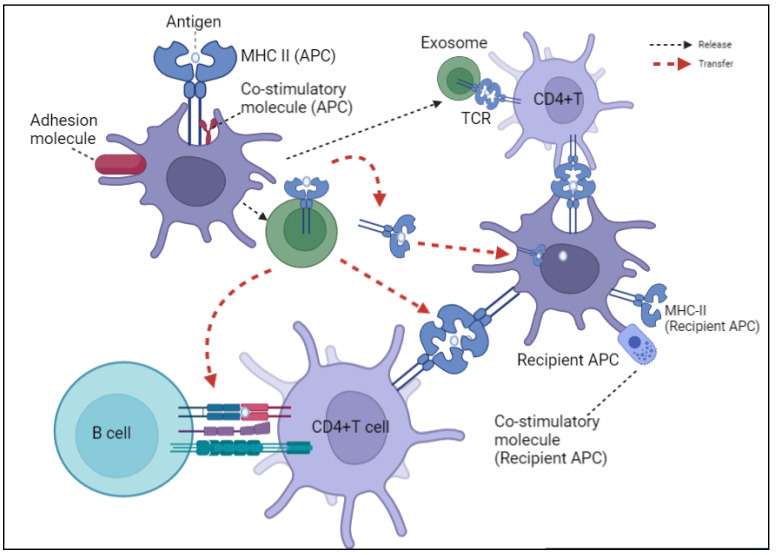
Role of exosomes in antigen presentation. Exosomes released by APCs (DCs) contain MHC II- antigen peptide complexes (MHC II-p) and can directly present antigen to CD4+T cell, deliver antigen to MHC II of recipient APC (red arrow), present antigen via cross-dressing (orange arrow), and transport MHC II-p to B cell (green arrow). Although only MHC II and CD4+ T cell are shown, exosomal MHC I undertakes a similar process in the regulation of CD8+ T cells.

EVs play a vital role in the regulation of the immune system, which may help prevent immune responses to various diseases [35]. The immune modulation of EVs may be attributed to the processing of antigen peptides and their presentation on their surfaces, followed by antigenic peptide transfer. As previously discussed [36], EVs contribute to antigen presentation via three primary mechanisms (Figure 1). The first mechanism involves the direct presentation of antigens, such as EVs derived from DCs, which bind to T cells directly via MHC-peptide complexes and costimulatory adhesion molecules. The second mechanism relies on indirect presentation of the antigen, in which EVs carrying antigenic peptides are transferred to MHC molecules of APCs, followed by T cell activation. The last mechanism is referred to as “cross-dressing,” in which the apprehended EVs are transferred to the surface of APCs and present their MHC-peptide conjugate directly to T cells for activation [36].

EVs enable the exploration of cell-free properties for modulating immune responses via MHC-complexed antigens to include tolerance of β-cells and autoreactive T cells. APCs secrete MHC-peptide conjugates that present transmembrane glycoprotein intercellular adhesion molecule-1 (ICAM-1) to EVs, which modulates the immune system via T cell activation. EVs secreted by APCs harbor functional peptide-MHC II and MHC I complexes as well as CD-80 and CD-86; these EVs might present antigens and initiate the activation of T lymphocytes [37]. EVs enhance T-cell-mediated responses and also contribute to the improvement of humoral responses, as they present native antigens that initiate the activation of B-lymphocytes [38]. In an immune-related exosome study using an animal model, EVs extracted from ovalbumin-loaded exosomes stimulated interferon-gamma (IFN-γ), which in turn activated humoral responses [39]. In another study, exosomes derived from B cells harbored peptide-MHC-II complexes that could enable prolonged antigen presentation to T cells [40].

A study involving mesothelioma patients reported that exosomes derived from pleural effusions containing transforming growth factor-beta (TGF-β) and NK group 2-member D (NKG2D) ligands inhibited CD8+ T cells and NK cells by downregulating the expression of NKG2D receptors [41]. In a similar study, placental cell-derived exosomes that possessed NKG2D ligands, such as UL-16 binding protein 1-5 (ULBP1-5) further modulated the surface expression of NKG2D on NK, CD8+, and γδ T cells, thereby downregulating their cytotoxic activity [35]. In another study, placental cell-derived exosomes containing the first apoptosis signal-ligand (FAS-L) could mediate apoptosis of CD4+ T cells [41].

Tolerogenic EVs play an important role in immune system modulation, which can be helpful in the management of autoimmune diseases. A recently published murine-model-based study reported that exosomes derived from DCs converted Th1/Th17 to Th2/Treg responses through miRNA-146a and potentiated the suppression of autoimmune myasthenia gravis (MG) [42]. Inflammatory bowel disease represents another autoimmune disease. It has been reported that DC-derived exosomes pretreated with soluble egg antigen can promote antigen tolerance and epithelial barrier function, thereby facilitating Treg expansion and Th1 cell proliferation inhibition [43]. Other authors reported that alpha-fetoprotein (AFP)-expressing, DC-derived exosomes possess anti-tumor activity mediated by the activation of IFN-γ-expressing CD8+ T cells with a simultaneous decrease in Tregs [44].

Exosomes derived from a unique group of CD4+CD25+Tregs were reported to protect against allograft rejection and aid in the prolonged survival of kidney transplant patients by suppressing T cell proliferation [45]. The mechanism of suppression by Treg-derived exosomes is still not fully understood; this effect may occur via the transfer of exosomal miRNAs to recipient cells [46]. In contrast, Treg-derived exosomes harboring CD73 can mediate the suppression of T cell proliferation [47]. Thus, immune-cell-derived exosomes can be engineered using various modification methods. Popular methods, including freeze–thaw, co-incubation, microfluidics, electroporation, and click chemistry, may permit the efficient modulation of the immune system, thereby targeting autoimmune diseases such as T1DM. Data from preclinical and clinical trials are required to support this hypothesis.

## 6. TNps and T1DM

The development of tolerance therapies requires precise identification and screening of antigenic targets of autoreactive immune cells, such as T cells. Small interfering ribonucleic acid (siRNA) has been used to inhibit the expression of chemokine receptor 2 (CCR2) [48]. Moreover, inhibition of chemokine receptors has been identified as a master regulator in the pathogenesis of diabetes mellitus, especially T1DM. In a clinical study, the CCR1/2 allosteric inhibitor reparixin improved outcomes during allogenic islet infusion and regulated islet damage [49]. An in vitro fabricated TNps of dextran-coated, iron oxide conjugated with siRNA downregulated MHC class I expression mediated by β2 microglobulin [50]. T1DM is a T-cell-mediated disease associated with MHC alleles. The primary role of MHC molecules is immune regulation through antigen presentation, especially in autoimmune diseases that include T1DM. Therefore, modulation of MHC-1 function using TNps has a significant therapeutic impact on the treatment approaches for T1DM. The immunosuppressive and anti-inflammatory characteristics of fabricated NPs in the regulation of the immune system are summarized in Table 2.

Shah et al. [51] fabricated a conjugate system with diblock polymer-based and rapamycin, and evaluated the system’s performance with an autoimmune disease. Rapamycin improves hepatic insulin sensitivity in patients with T1DM [52]. Therefore, engineering approaches using rapamycin are important for the fabrication of TNps for the treatment of autoimmune diseases, such as T1DM. Furthermore, iron oxide NPs with peptide conjugate systems are tolerogenic in MHC I and II modulation in autoimmune disorders [53]. Iron oxide NPs are not toxic to human health because upon degradation of these NPs, the contents are processed via natural iron metabolism pathways. There are few side effects and negligible suppression of immune function [53].

In another tolerogenic approach, gold NPs (AuNPs) were used with polyethylene glycol (PEG) to modulate T cell epitopes via the uptake of these NPs by DCs. This mechanism was demonstrated to expand Foxp3+ Tregs, further reducing the severity of experimental autoimmune encephalomyelitis [54]. These NPs were further evaluated in a murine NOD model for the treatment of T1DM [55]. The possible mechanism of these AuNPs in T1DM relies on the induction of tolerogenic responses in DC by AuNPs via induction of the suppressor of cytokine signaling 2 (Socs2), which results in the inhibition of nuclear factor κB (NF-κB) activation and proinflammatory cytokine production [55]. T cells can be evaluated to determine their potential use in the treatment of autoimmune diseases, particularly T1DM. Serr et al. suggested the development of targeted therapy for T1DM islet autoimmunity using miRNA181a and/or nuclear factor of activated T cells-5 (NFAT5) signaling [56]. Increasing miRNA181a activity boosts NFAT5 activity, while inhibiting FOXP3+ Treg induction [56]. Tolerogenic iron oxide NPs surface engineered with the proinsulin auto-antigen and 2-(1′H-indole-3′-carbonyl)-thiazole-4-carboxylic acid methyl ester (ITE) can be used for the early diagnosis of T1DM when employed with magnetic resonance imaging combined with magnetic quantification [57].

Use of antisense oligonucleotides to CD40, CD80, and CD86 suppresses DC activation [58]. The infusion of autologous DCs pretreated with antisense nucleotides can significantly delay the progression of T1DM [58]. However, no significant changes in immunological function were observed in phase I clinical trials of T1DM patients receiving these pretreated DC infusions [58]. Phillips et al. [59] encapsulated antisense oligonucleotides into synthetic microspheres (microparticles) and injected them s.c. The microparticles were taken up by DCs, which exhibited a suppressive phenotype downregulating the expression of T-cell-activating modulators. The downregulation significantly reverted hyperglycemia in a murine NOD mouse model. T1DM is mediated by the CD8+ T cell immunological mechanism; thus, the delivery of autoantigens and simultaneous release of TNps may promote the immunomodulation of CD8+ T cells. In another study, the delivery of cognate peptide antigen encapsulated within poly(lactic-co-glycolic acid) NPs could re-educate immunity toward a tolerant state, as demonstrated in a transgenic T1D mouse model [60]. Spontaneous death of β-cells in T1DM is attributed to DC activation [7]. The application of such TNps for the modulation of DCs has several advantages relative to the conventional approach of DC modulation. These include: (i) providing immunity to antigen cargo from protease action, (ii) enabling co-delivery of several NPs using EVs as vehicles, (iii) providing controlled release and delivery, and (iv) reducing non-specific target recognition. These tolerogenic biological NPs provide a safer therapeutic approach and are potentially valuable in the treatment of T1DM.

## 7. Methods to Fabricate Engineered Tolerogenic EVs with NPs

Tolerogenic EVs exhibit lower loading efficiency due to their small size. However, these EVs have low toxicity and tend to diffuse through the basement membrane. Thus, tolerogenic EVs could be a promising therapeutic approach [61,62]. EVs are composed of several biological molecules, including membrane proteins and lipid bilayers. The modification potential of EVs may potentially be exploited for the delivery of drugs and other TNps for the treatment of various autoimmune diseases. There are two engineering-based approaches available for the modification of EVs: engineering of EV-secreting cells and post-isolation engineering.

### 7.1. Production of EVs by Cell Engineering

Cells secreting EVs can be engineered by two approaches: culture of cells in media/environments that impose stresses including hypoxia, serum starvation, and inflammation [63,64,65] and transfection of cultured cells with modulators that include plasmid DNA, miRNAs, miRNA antagonists, and Y RNA. In a cardio-protective study, EVs derived from progenitor cells grown under hypoxic conditions displayed an increased tendency to form tube-like entities compared to EVs derived under normoxic conditions [66]. Cells secreting EVs can be modulated by changing the culture medium. EVs derived from human adipose stem cells cultured in differential endothelial medium showed increased levels of miRNA-31 [67]. Modulated cells by external agents via transfection is also possible. For example, in one study miRNA181a was transfected into human mesenchymal stem cells, which led to a significant pro-reparative state in peripheral blood mononuclear cells [68]. The advantages and associated limitations of EV modification are summarized in Table 3.

A genetic engineering approach to perform modifications to enable EV delivery of novel payloads is also a popular method among researchers. Alvarez-Erviti et al. [69] used genetic engineering to perform delivery of siRNA using EVs. The main advantage of using this approach is that it always generates a homogenous population of EVs without toxicity. The versatile method allows the loading of RNA, DNA, and peptides of choice into exosomes. Limitations of this method include the choice of donor cells for harvesting exosome populations. Tumor-derived exosomes may interact in the pre-metastatic niche and initiate negative effects. The current number of studies thus far are insufficient to determine the negative effects of exosomes derived from tumor cells. The use of adenoviral genes is also a limiting factor, since in some cases humans have developed an immune response that limits gene expression. In another study, curcumin-loaded mouse lymphoma EL-4 exosomes were synthesized by simple mixing of curcumin and exosomes. Thereafter, the anti-inflammatory activity of the synthesized exosomes was investigated in a murine model of lipopolysaccharide (LPS)-mediated septic shock [70].

### 7.2. Post-Isolation Engineering

Post-isolation engineering provides a better approach for modulating EVs and preserving their biological content and function. This method incorporates bioengineering of EV loading, targeting, and delivery into target cells. EV-modulating strategies include passive loading (incubation with EVs and donor cells) and active loading (extrusion, freeze–thawing, electroporation, sonication, chemical transfection, Click Chemistry, and antibody binding) [71]. EVs have been modified and loaded with curcumin using an incubation approach; modified versions of these anti-inflammatory EVs were used to treat LPS-induced septic shock [70]. In this study, physical entrapment and chemical conjugation were used to load NPs with curcumin [70]. The hydrophobic nature of EVs due to the presence of a lipid bilayer enabled the easy incorporation of hydrophobic curcumin into NPs that self-reassembled, with size-dependent selective distribution evident among tissues [70]. Kim et al. used the simplest reported co-incubation method to incorporate drugs into EVs [72].

EVs can be enriched using an electroporation approach for a short period of time. This approach enables drugs or NPs to easily penetrate the double-layered lipid membrane of EVs under the application of an electric field. Drug loading of EVs using electroporation has been described [72]. In another study, EVs derived from DCs were loaded with doxycycline using an electroporation approach to inhibit tumor growth [73]. In another approach for the modification of EVs, the sonication method (6 cycles of 30 on/off for 3 min, followed by a 2 min cooling period) was used to load drugs into EVs [72]. EVs derived from cardiac progenitor cells were also enriched with miRNA-322 by electroporation to treat myocardial infarction [74]. Furthermore, the EV surface was modified using streptavidin and peptides via linkers attached to the carboxylic and amine groups following copper-free click chemistry [75].

Engineering of cells and post-isolation engineering of EVs have advantages and disadvantages. The genetic engineering approach produces standardized controlled EVs with desired traits. However, limitations include alterations in the biological activities of EVs due to the desired gene transfection and uncontrollable density of modification (number of epitopes attached per surface area of the EV). Limitations of post-isolation engineering include low yield, use of harsh chemicals that disrupt the EV composition, heat produced by the electric field, and sonication. These approaches should be used in a controlled manner to preserve the structure and function of the particular EVs.

## 8. Role of Post-Engineered EVs with TNps in T1DM

Several engineering-based strategies have been developed to fabricate tolerogenic EVs containing NPs for the treatment of T1DM. Immunomodulatory NPs or microparticles carrying antisense oligonucleotides to CD40, CD80, and CD86 were delivered to NOD mice, and T1DM was prevented by augmentation of Foxp3+ Treg cells [59]. In a murine model of diabetes mellitus, co-culture of islets and bone marrow stem cells increased survival and functionality of islet β-cells mediated by exosomes through a paracrine effect [76]. A clinical study also revealed the role of exosomes derived from mesenchymal stem cells in the suppression of immune targeting in allogeneic grafts [77]. The findings indicate that EVs are beneficial in islet β-cell restoration because of their regenerative, anti-apoptotic, immunomodulatory, and angiogenic properties.

The delivery of antisense oligonucleotides against the primary transcripts of APC costimulatory molecules (CD40, 80, and 86) can inhibit DC activation and has been implicated as a preventive therapy for diabetes [78]. This approach can easily be translated further via the delivery of antisense oligonucleotides mediated by EVs using a genetic engineering approach. Another study revealed delayed progression of T1DM via the delivery of antisense nucleotide-treated autologous DCs [79]. The findings can also be further translated into a genetic engineering approach via the modification of autologous DCs with antisense nucleotides in the culture medium. Subcutaneous injection of microparticles incorporated with antisense oligonucleotides reversed hyperglycemia in a diabetic murine model [79,80]. These oligonucleotides could be further explored via EV-based delivery using post-isolation engineering approaches for the treatment of autoimmune T1DM. TNps of poly (lactic-co-glycolic acid) have also been functionalized with anti-CD4 and interleukin-2 for targeting the T cell response [81].

Tolerogenic NPs targeting specific antigens cause specific immunosuppression, which may effectively treat T1DM by restoring T cell immune tolerance. The methyl ester of 2-(1Hindole-3-carbonyl)-thiazole-4-carboxylic acid (HCTCAME) has been used as a tolerogenic agent to increase DC activation [82]. Similarly, the β-cell antigen proinsulin was adsorbed on the surface of AuNPs for T1DM management [83]. Alternatively, β-cell antigen, proinsulin, and HCTCAME can be effectively explored via their incorporation into EVs using post-isolation engineering methods that may produce multiple therapeutic effects in T1DM. EVs can provide an infinite supply of cellular NPs that can modulate immune functions in an immunostimulatory or immunoregulatory manner, and induce antigen-specific tolerance of β-cell autoreactive T cells, particularly in T1DM. A recently published study related to T1DM showed that EVs derived from mesenchymal stem cells prevent T1DM onset and activate Th1 and Th17 cells [84]. Several risk factors and immune components are involved in the pathogenesis of T1DM. Therefore, anti-aging biomaterials, TNps, and EVs that modulate immune functions would be beneficial for the prevention and treatment of T1DM.

## 9. Factors Affecting Tuning of TNps to Boost Immunomodulation

Boosting targeted immunomodulation using TNps requires the optimization of several physicochemical measures. These factors are useful for boosting immune responses and are also responsible for maintaining the quality of responses within the cells. The use of engineered nanoparticles presents a better approach for cargo loading [85,86]. Numerous potential materials can be used in the fabrication of immune cell-targeting NPs to modulate immune responses. These materials must meet the primary requirements of biocompatibility, non-toxicity, and ease of modification of shape, surface chemistry, and size to enable effective results. Liposomes as an organic starting material have been employed in two published studies for the fabrication of nano-formulation [87,88]. In one of these studies, phosphatidylserine-liposomes (PL) loaded with insulin peptides were fabricated to stimulate apoptotic cells for detection by APCs. The PL were used in a spontaneous mouse model of autoimmune diabetes [88]. The authors reported that PL containing insulin peptides activated tolerogenic DCs, thereby impairing autoreactive T cell proliferation.

The optimum size of NPs plays a significant role in boosting and tuning the immune response. The decreased surface-to-volume ratio of larger TNps affects their interactions with immune cells. NP uptake includes four endocytic aspects: pinocytosis, macropinocytosis, phagocytosis, and clathrin/caveolar-mediated endocytosis; immune cells commonly adopt pinocytosis and micropinocytosis mechanisms [89]. Table 4 summarizes the importance of the material and its size in the reversal of T1DM. Moreover, the shape of engineered NPs significantly affects the immune modulation mechanism [90]. Gold-based nanorods reportedly display efficient uptake by macrophage cells compared to nanospheres [91]. Furthermore, the size and shape of the engineered TNps contribute to the tuning and boosting of immune modulation during T1DM therapy.

Several factors associated with NPs affect their uptake and interaction with immune components. Adjusting these factors is crucial for fabricating engineered NPs for immune modulation. The surface chemistry of NPs, such as charge and hydrophilicity/hydrophobicity, is another avenue for significant interaction with immune cells. In a previously published study, it was demonstrated that nanoparticles coated with peptides upon systemic delivery showed binding with MHC II that further activated the expansion of CD4(+) T cell type 1 in rodent models and proved the positive modulating role in autoimmune mechanism [95]. Similarly, another study reported the utility of T-lymphocyte-derived exosomal micro-RNAs (miRNAs) including miR-142-3p, miR-142-5p, and miR-155 in initiating the β cells apoptosis, which can be further used as therapeutic targets [96,97]. T1DM is mostly not associated with comorbidities such as micro- and macrovascular complications including diabetic retinopathy, nephropathy, neuropathy and cardiovascular diseases. However, T2DM is frequently presented with such complications [97]. EVs have not only demonstrated a therapeutic immunomodulatory role in T1D, but also showed promising treatment regime to patients with T2DM having such complications. On the contrary, one previously published study reported that EVs can induce insulin resistance, thereby contributing to development of T2DM through uncontrolled hyperglycemia [97]. However, a similar study also proved the therapeutic role of EVs in T2DM. The authors of previously published studies showed that EVs can act as therapeutic targets in T2DM patients with cardiovascular disease [98,99]. The pathophysiology of such therapeutic effects is mediated by mi-RNAs as suggested by such studies [100]. NPs with highly dense positive or negative charges exhibit colloidal stabilization owing to the electrostatic repulsive forces. NPs with a positive surface charge were efficiently internalized by cells and exhibited high immunogenic potential [101]. Some of the relevant studies also establish the role of immune cells derived EVs as therapeutics [102]. Notably, these factors contribute to immune response modulation during the fabrication of engineered TNps.

## 10. Conclusions

Cellular uptake of engineered EVs containing TNps could provide the basis of a treatment strategy for T1DM. The characteristics of these TNps and EVs, including size, shape, and ease of systemic circulation, significantly modulate immune cell function. Numerous engineering approaches for EV modification, including genetic and post-isolation approaches, provide an opportunity for their translation into tolerogenic EVs without the limitations associated with drug-based or cell-based therapies in T1DM. EVs may present a safer approach for the treatment of T1DM than chemical-based interventions. However, more preclinical and clinical trials are required to support the statements mentioned in this review.

## Figures and Tables

**Table 1:** Classification of EVs Based on Their Size, Density, and Mode of Biogenesis.

EV Type	Diameter (nm)	Density (g/mL)	Cell Type Origin	Subcellular Origin
Exosomes	30–150	1.13–1.19	Most cell types	MVB
	<100	1.10–1.18		
Microvesicles	200–1000	1.04–1.07	Most cell types	PM-shed vesicle
	100–1000			
Apoptotic bodies	1000 to >5000	1.16–1.28	All cell types	PM-shed vesicle
	500–4000			
Large oncosomes	1000–10 000	1.10–1.15	Tumor cells	PM-shed vesicle

Data are adopted from [27] under Creative Commons Attribution 4.0 License.

**Table 2:** Direct and indirect immunosuppressive and anti-inflammatory properties of NPs.

	Mechanism of Action	
	Indirect	Direct
**Anti-inflammatory**	**Carriers for anti-inflammatory drugs** (corticosteroids, indomethacin, methotrexate) liposomes, dendrimers, polymeric NPs	**Inhibition of COX and proinflammatory signaling** polyamidoamine dendrimers, gold NPs nanoparticles
	**Carriers for anti-cytokine agents** (receptors’ antagonists, siRNA against cytokines and signaling molecules, DNA of anti-inflammatory cytokines) polymeric NPs, dendrimers, liposomes, chitosan NPs	**Antioxidant activity** (cerium oxide NPs, gold NPs, fullerene derivate)
	**Anti-adhesion agents** (siRNA against CCR2, selectin antagonists) lipid, NPs, dendrimer-like polymers	**Anti-cytokine activity** gold NPs
**Immunosuppressive**	**Carriers for traditional immunosuppressive drugs** (cyclosporine, tacrolimus, rapamycin, mycophelic acid) liposomes, polymeric NPs, lipid NPs	**Inhibition of T-cell-mediated immunity** Iron oxide NPs, fullerene 60
	**Tolerogenic vaccines** (antigens, costimulatory signals) polymeric NPs, iron oxide NPs, polyethylene glycol-gold NPs, chitosan NPs	**Interference with functions of immune system cells** Iron oxide NPs, *polyvinyl alcohol-superparamagnetic iron oxide NPs*, multi-walled carbon nanotubes, quantum dots
	**Myelosuppression** (increase toxicity of a carried drug) polyisobutylcyanoacrylate, polyisohexylcyanoacrylate	**Myelosuppression and toxicity to cells of the immune system** Sb_2_O_3_, Co, ZnO, TiO_2_ NPs

**Table 3:** Post-isolation strategies for EV modification.

	Modification Strategies	Advantages	Disadvantages
**Passive loading**	Incubation of exosomes and free drugs	Simple	Incubation of exosomes and free drugs
	Incubation of donor cells with free drugs	Simple	Incubation of donor cells with free drugs
**Active loading**	Sonication	High drug-loading efficiency	Compromised membrane integrity
	Extrusion	High drug-loading efficiency	Compromised membrane integrity
	Freeze/thaw	Medium drug-loading efficiency	Freeze/thaw damage
	Electroporation	Loading with large molecules, such as siRNA and miRNA	Aggregation
	Incubation with saponin	Enhanced drug loading	Toxicity
	Click chemistry	Quick and efficient	None
	Antibody binding	Specific and easy to operate	None

The data are adapted from [71] under Creative Commons Attribution 3.0 License.

**Table 4:** Engineered NPs of varying size for the reversal of autoimmune T1DM.

Biomaterial	Size of Engineered NPs (nm)	Cargo of Interest Loaded into NPs	References
PSL	1000	Insulin A and B peptides	[88]
Gold	60	ITE and proinsulin	[92]
PLGA	1000	Vitamin D3 and insulin B9–23 (1 µm)	[93]
PLGA	30,000	GM-CSF and TGF-β1 (30 µm)	[93]
PLGA	1000	Antisense RNA for CD40, CD80	[94]
PLGA	1800	CD86 oligonucleotides	[94]

ITE = 2-(1H-indole-3-carbonyl)-thiazole-4-carboxylic acid methyl ester; PLGA = poly (lactic-co-glycolic acid); PSL = phosphatidylserine-liposomes.

## Data Availability

Available on request to the corresponding author.
